# Biopsy proportion of tumour predicts pathological tumour response and benefit from chemotherapy in resectable oesophageal carcinoma: results from the UK MRC OE02 trial

**DOI:** 10.18632/oncotarget.12723

**Published:** 2016-10-18

**Authors:** Matthew D. Hale, Matthew Nankivell, Gordon G. Hutchins, Sally P. Stenning, Ruth E. Langley, Wolfram Mueller, Nicholas P. West, Alexander I. Wright, Darren Treanor, Lindsay C. Hewitt, William H. Allum, David Cunningham, Jeremy D. Hayden, Heike I. Grabsch

**Affiliations:** ^1^ Pathology and Tumour Biology, Leeds Institute of Cancer and Pathology, University of Leeds, Leeds, UK; ^2^ Medical Research Council Clinical Trials Unit at University College London, London, UK; ^3^ Department of Histopathology, St James's Institute of Oncology, Leeds Teaching Hospitals NHS Trust, Leeds, UK; ^4^ Gemeinschaftspraxis Pathologie, Starnberg, Germany; ^5^ The Royal Marsden Hospital NHS Foundation Trust, London and Surrey, UK; ^6^ Department of Upper Gastrointestinal Surgery, St James's Institute of Oncology, Leeds Teaching Hospitals NHS Trust, Leeds, UK; ^7^ Department of Pathology, GROW School for Oncology and Developmental Biology, Maastricht University Medical Centre, Maastricht, Netherlands

**Keywords:** oesophageal cancer, proportion of tumour, neoadjuvant chemotherapy, biomarker, tumour stroma

## Abstract

**Background:**

Neoadjuvant chemotherapy followed by surgery is the standard of care for UK patients with locally advanced resectable oesophageal carcinoma (OeC). However, not all patients benefit from multimodal treatment and there is a clinical need for biomarkers which can identify chemotherapy responders. This study investigated whether the proportion of tumour cells per tumour area (PoT) measured in the pre-treatment biopsy predicts chemotherapy benefit for OeC patients.

**Patients and methods:**

PoT was quantified using digitized haematoxylin/eosin stained pre-treatment biopsy slides from 281 OeC patients from the UK MRC OE02 trial (141 treated by surgery alone (S); 140 treated by 5-fluorouracil/cisplatin followed by surgery (CS)). The relationship between PoT and clinicopathological data including tumour regression grade (TRG), overall survival and treatment interaction was investigated.

**Results:**

PoT was associated with chemotherapy benefit in a non-linear fashion (test for interaction, *P=*0.006). Only patients with a biopsy PoT between 40% and 70% received a significant survival benefit from neoadjuvant chemotherapy (*N=*129; HR (95%CI):1.94 (1.39-2.71), unlike those with lower or higher PoT (PoT<40%, *N=*39, HR:1.25 (0.66-2.35); PoT>70% (*N=*28, HR:0.65 (0.36-1.18)). High pre-treatment PoT was related to lack of primary tumour regression (TRG 4 or 5), *P=*0.0402.

**Conclusions:**

This is the first study to identify in a representative subgroup of OeC patients from a large randomized phase III trial that the proportion of tumour in the pre-chemotherapy biopsy predicts benefit from chemotherapy and may be a clinically useful biomarker for patient treatment stratification.

## Abstract 2

Proportion of tumour is a novel biomarker which can be measured in the pre-treatment diagnostic biopsy and which may enable the identification of chemotherapy responders and non-responders among patients with oesophageal carcinoma. Proportion of tumour could easily become part of the routine reporting of oesophageal cancer biopsies and may aid in managing patients with borderline resectable cancer.

## INTRODUCTION

Oesophageal cancer (OeC) is the 6th commonest cause of cancer death worldwide, accounting for approximately 400,000 cancer-related deaths in 2012 [1]. At the time of diagnosis, OeC is potentially resectable, and thus potentially curable, in 30% of patients. The cornerstone of curative OeC treatment is surgical resection preceded by neoadjuvant combination chemotherapy (NAC) or chemoradiotherapy to downstage the disease, enable complete resection and eliminate micrometastases preventing recurrent metastatic disease [2–6].

Only a relatively small subset of patients with resectable OeC appear to have long-term benefit from multimodality treatment whereas chemotherapy non-responders may have an unnecessarily prolonged wait for surgery with potential tumour progression and toxic side effects [6, 7]. In order to optimize treatment for OeC patients, there is an urgent clinical need to identify biomarkers that predict local tumour response and longterm chemotherapy benefit, ideally using the routine diagnostic endoscopic biopsy material.

Like most other malignant tumours, OeC are composed of tumour cells and intratumoural stroma (extracellular matrix, fibroblasts, vessels, immune cells *etc.*). Intratumoural stroma varies in quantity and quality and has been shown to influence malignant transformation, tumour invasion and metastasis (for review see Hanahan *et al*. [8]). Stroma abundance has been related to poor patient prognosis [9–22]. Intratumoural stroma has also been associated with chemotherapy resistance by fibroblast proteins inhibiting tumour cell apoptosis [23] or by reducing chemotherapy delivery [24]. To date, there have been no studies published investigating the role of the proportion of tumour (PoT), i.e. the relative amount of tumour cells and intratumoural stroma in the tumour area, in predicting local tumour response and survival benefit from cytotoxic combination chemotherapy.

This study tested the hypothesis that high PoT (e.g. low intratumoural stroma content) in the pre-treatment biopsy predicts benefit from neoadjuvant chemotherapy in OeC patients. PoT was quantified in the routine diagnostic pre-treatment endoscopic biopsy slides from OeC patients randomized to treatment with either surgery alone (S group) or chemotherapy followed by surgery (CS group) within the UK MRC OE02 trial (ISRCTN 43987580) [2]. The relationship between PoT and clinicopathological data including pathological tumour regression grade, overall survival and treatment interaction effect was investigated.

## RESULTS

### Patients and clinicopathological data

**Figure 1:**
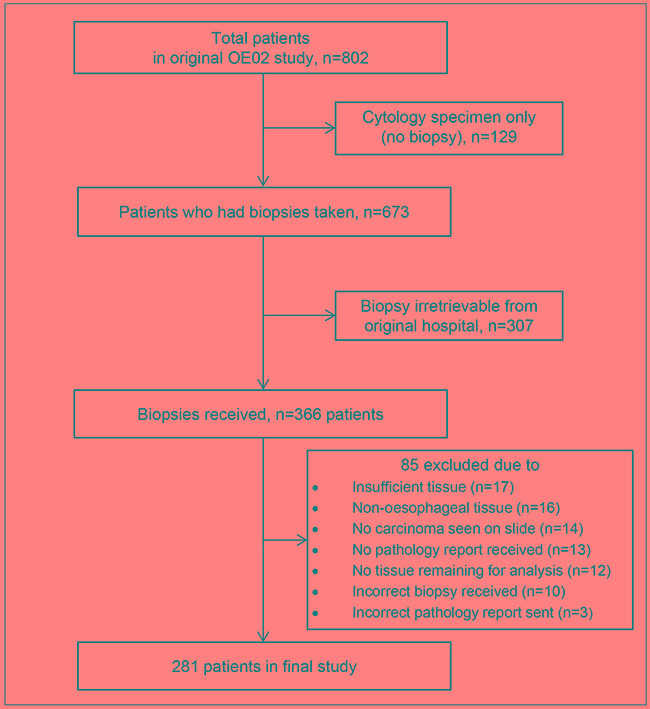
Consort diagram showing the details of biopsy material retrieval from the OE02 trial patients

Biopsy material from 281 OE02 trial patients (140 patients from the chemotherapy followed by surgery (CS) group and 141 patients from the surgery alone (S) group) was included in the current study (details of material retrieval and case drop outs are shown Figure 1). Patient median age was 62 years (range: 30 to 83 years). Two hundred and thirteen patients (76%) were male, 195 patients (69%) had adenocarcinoma, 84 patients (30%) had squamous cell carcinoma and 2 patients (1%) had undifferentiated carcinomas. Baseline characteristics of the patients included in the study were similar between the two treatment arms and similar to the whole OE02 trial cohort with respect to survival (HR=0.97 95%CI: 0.83-1.14, *P=*0.72) and clinicopathological data (Supplementary Table S1).

### Inter-observer variation of proportion of tumour measurement

Double scoring of 10% of tissue pieces (48 475 measurement points) demonstrated 95.54% agreement for individual measurement points between the two observers (κ=0.937, 95% confidence interval (CI): 0.935 to 0.938, *P*<0.0001).

After calculating the PoT per piece for each observer and using the methodology described by Bland-Altman, [25] the mean difference in PoT between the two observers was 2% (95%CI:1.3 to 2.8%).

### Relationship between proportion of tumour and clinicopathological data

In total, 838 tissue pieces from 281 patients were measured and a total of 498,497 measurement points were categorized. The median number of tissue pieces per patient was 5 (range: 1 to 20 pieces). The median number of tissue pieces meeting the inclusion criteria for analysis (see methods) was 3 (range: 1 to 12 pieces). The median size of the region of interest per patient was 1.44 mm^2^ (range: 0.04 mm^2^ to 23.2 mm^2^).

The median proportion of tumour (PoT) per patient was 56% (range: 15% to 100%), 85% of patients had a PoT between 25% and 75%. PoT in the pre-treatment biopsy was not different between the CS group and the S group (CS median PoT: 56% (range: 17 to 89%); S median PoT: 55% (range: 15 to 99%), *P=*0.467). PoT of squamous cell carcinoma biopsies was significantly higher than that of adenocarcinoma biopsies (squamous cell carcinoma median PoT: 58% (range: 17 to 93%); adenocarcinoma median PoT: 55% (range: 15 to 99.5%), *P=*0.0357). When analyzing the data of the whole patient cohort, there was no relationship between PoT and age (*P=*0.3096), gender (*P=*0.6571), tumour location (*P=*0.6391), grade of differentiation (*P=*0.7409), degree of dysphagia (*P=*0.0626) or World Health Organization performance status (*P=*0.6382). Representative images of the PoT groups (PoT <40%, 40-70% and >70%) are shown in Supplementary Figure S1. The associations between the PoT categories and the clinicopathological variables are shown in Supplementary Table S2.

Investigating the patients with biopsy and matched resection specimens (*N=*111), there was no significant relationship between biopsy PoT (classified as PoTlow, PoTmedium, PoThigh, see below) and depth of invasion (T category) or lymph node status (N category) neither for CS patients (*P=*0.2722 and *P=*0.5021, respectively) nor for S patients (*P=*0.1570 and *P=*0.8851, respectively).

### Relationship between proportion of tumour in the pre-treatment biopsy and Mandard tumour regression grade in the resected specimen

Matched resection specimens to determine Mandard tumour regression grade (TRG) were available for 111 (79%) of the 140 CS patients with PoT data. Evidence of tumour regression, defined as Mandard TRG 1, 2 or 3, was seen in 18 (16%) OeC. When analysed as a continuous variable, PoT in the pre-treatment biopsy was significantly higher in cases with no evidence of tumour regression (TRG 4 or 5) (median PoT: 57% (range: 17 to 86%)) compared to those with evidence of tumour regression (TRG 1, 2 or 3) (median PoT: 52%, (range: 25 to 75%)), *P=*0.0402, Supplementary Figure S2.

### Relationship between proportion of tumour in the pre-treatment biopsy and patient survival

**Figure 2:**
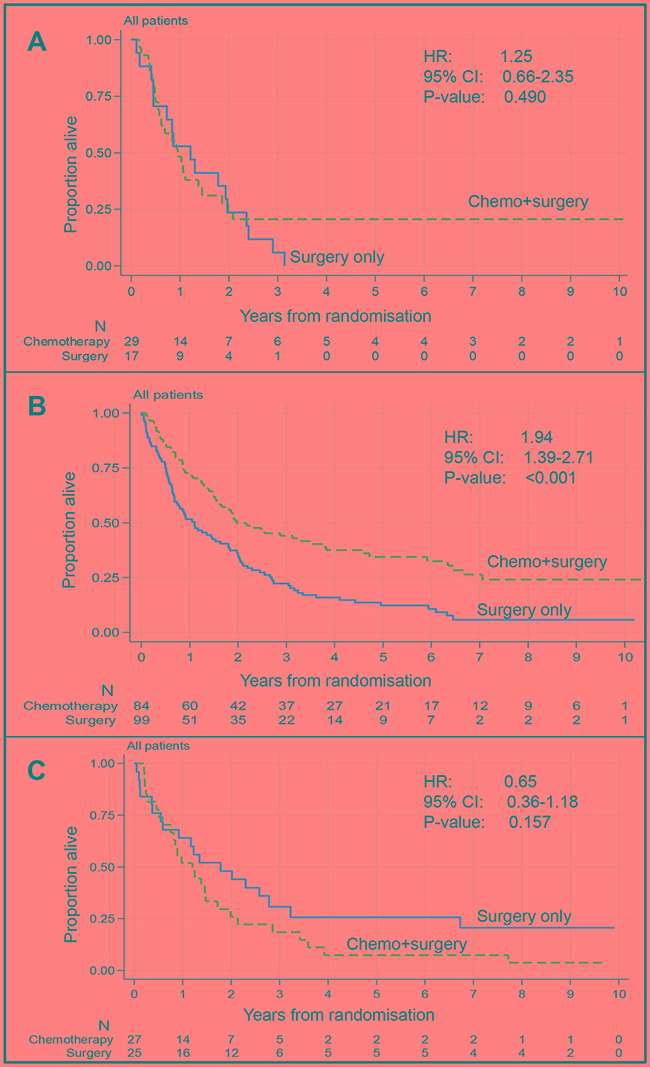
Relationship between treatment and overall survival by proportion of tumour class (whole cohort) **A.** Proportion of tumour < 40%. There is no significant difference in overall survival between patients treated with chemotherapy followed by surgery (n=29) compared to those treated by surgery alone (n=17). **B.** Proportion of tumour between 40% and 70%. Patients treated with chemotherapy followed by surgery (n=84) survived significantly longer than patients treated with surgery only (n=99). **C.** Proportion of tumour > 70%. There is no significant difference in overall survival between patients treated with chemotherapy followed by surgery (n=27) compared to those treated by surgery alone (n=25).

The prognostic value of PoT is summarized in Table 1. Among patients treated with surgery only, there was a trend that patients with a higher PoT had a longer survival (log-rank trend *P*-value 0.0359). For CS patients, a non-linear association between PoT and patient survival was seen. CS patients with PoTmed (≥ 40% ≤ 70%) tumours had a significantly longer overall survival than those with PoTlow (<40%) or PoThigh (>70%) tumours with evidence of a treatment interaction effect (*P*-value of test for heterogeneity = 0.006), see Table 1. When comparing treatment arms, only patients with PoTmed in the pre-treatment biopsy demonstrated a significant survival benefit from chemotherapy (HR 1.94 (95%CI: 1.39-2.71, *P*-value<0.001), Figure 2 and Table 2. No difference in overall survival was seen between CS patients and S patients with PoTlow tumours (HR 1.25, 95%CI: 0.66-2.35, *P*=0.490) or PoThigh tumours (HR 0.65, 95%CI: 0.36-1.18, P=0.157), suggesting that OE02 trial patients with a PoThigh tumour in the diagnostic biopsy may benefitted most from treatment with surgery alone.

**Table 1:** Prognostic value of proportion of tumour by treatment arm

PoT grouping	Pre-op chemotherapy followed by surgery			Surgery alone		
	*N*	Median OS	HR (95% CI)	*N*	Median OS	HR (95% CI)
**Continuous**	140		1.00 (0.99, 1.01)	141		0.99 (0.98, 1.00)
**Binary**						
<50%	51	1.25	1	49	1.45	1
≥50%	89	1.85	0.83 (0.56, 1.23)	92	1.10	0.93 (0.65, 1.34)
**Tertiles**						
≤49.15	47	1.11	1	47	1.45	1
49.16 – 64.03	49	1.92	0.71 (0.45, 1.13)	46	1.05	1.10 (0.72, 1.68)
>64.03	44	1.46	0.92 (0.59, 1.46)	48	1.11	0.84 (0.54, 1.30)
**Final grouping**						
<40	29	0.95	1*	17	1.21	1**^†^**
**40-70**	**84**	**1.98**	0.59 (0.37, 0.97)*	99	1.10	0.75 (0.44, 1.27)**^†^**
≥70	27	1.20	1.20 (0.69, 2.11)*	25	1.78	**0.50 (0.26, 0.97)^†^**

Values of significance are highlighted and typed in bold. Abbreviations: CI: confidence interval: HR: hazard ratio; OS: overall survival (years); PoT: proportion of tumour.

* test for interaction of heterogeneity of treatment effect across groups, *P*=0.006

† log-rank trend *P*-value=0.0359

**Table 2:** Predictive value of proportion of tumour

PoT grouping	Pre-op chemotherapy followed by surgery		Surgery alone		HR (95% CI)
	*N*	Median OS	*N*	Median OS	
**PoTlow (<40%)**	29	0.95	17	1.21	1.25 (0.66 - 2.35)
**PoTmed (40-70%)**	**84**	**1.98**	**99**	**1.10**	1.94 (1.39 - 2.71)*
**PoThigh (>70%)**	27	1.20	25	1.78	0.65 (0.38 – 1.18)

**Figure 3:**
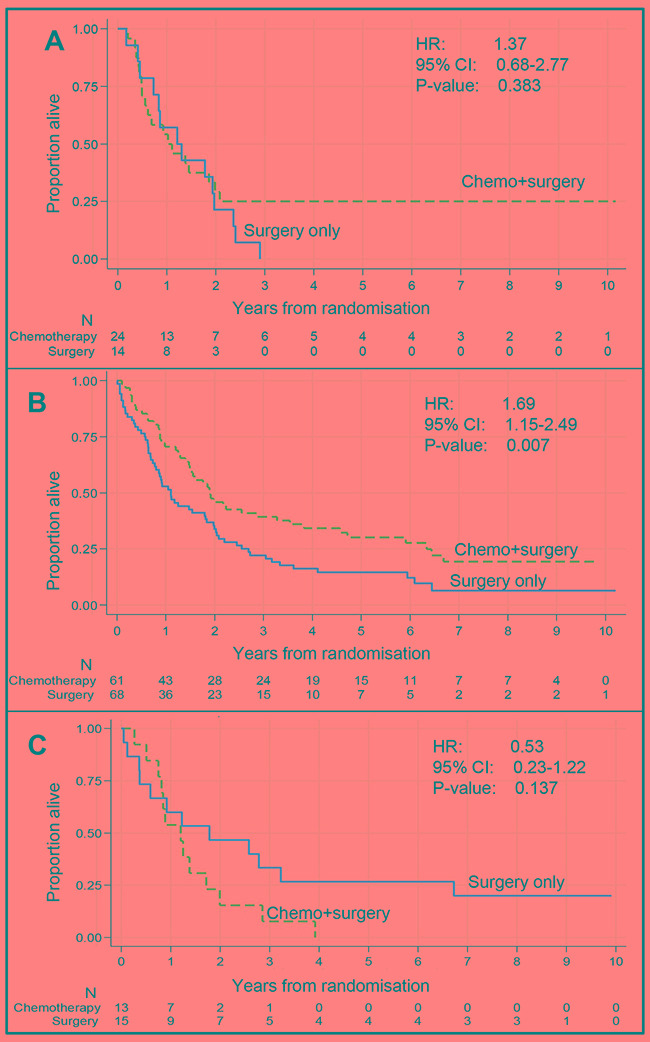
Relationship between treatment and overall survival by proportion of tumour class (adenocarcinoma patients only) Relationship between treatment and overall survival by proportion of tumour class (adenocarcinoma patients only) **A.** Proportion of tumour < 40%. There is no significant difference in overall survival between adenocarcinoma patients treated with chemotherapy followed by surgery (n=24) compared to those treated by surgery alone (n=14) **B.** Proportion of tumour between 40% and 70%. Adenocarcinoma patients treated with chemotherapy followed by surgery (n=61) survived significantly longer than patients treated with surgery only (n=68). **C.** Proportion of tumour > 70%. There is no significant difference in overall survival between adenocarcinoma patients treated with chemotherapy followed by surgery (n=13) compared to those treated by surgery alone (n=15).

The relationship between PoTlow, PoTmed and PoThigh and survival was similar when the analyses were restricted to the subgroup of patients with adenocarcinoma (CS: *N*=98, S: *N*=97). Among adenocarcinoma patients with PoTmedium biopsies, a longer survival was seen for CS patients with evidence of a treatment interaction effect (*P*-value of test for heterogeneity = 0.046), Supplementary Table S3. As with the full cohort, only patients with PoTmed biopsies demonstrated a significant survival benefit from treatment with neoadjuvant chemotherapy (Figure 3). No significant difference in survival and no treatment interaction effect was observed among adenocarcinoma patients with PoThigh or PoTlow tumours (Figure 3).

## DISCUSSION

This is the first study to investigate whether the proportion of tumour (PoT) in the pre-treatment endoscopic biopsies can predict local tumour response and benefit from neoadjuvant combination chemotherapy in patients with oesophageal cancer (OeC) from the randomized UK MRC OE02 phase III trial. Using virtual slides and quantitative morphometry with near perfect inter-observer agreement, we demonstrated that the biopsy PoT is able to identify patients who benefitted from chemotherapy. However, to our surprise, the relationship between PoT and survival was not linear. Only patients with a PoT between 40% and 70% derived a significant benefit from neoadjuvant chemotherapy. Furthermore, comparison between the two treatment arms suggests that for patients with a very high PoT, survival following treatment with neoadjuvant chemotherapy may be poorer compared to treatment by surgery alone. To date, no studies have performed a similar investigation on clinical material in either oesophageal cancer or in any other cancer types treated with neoadjuvant chemotherapy. None of the previous studies, which investigated PoT as a prognostic marker, identified a non-linear relationship between PoT and survival. This could be related to the fact that previous studies didn't use quantitative morphometry methods which generate continuous data that can be explored in detail or that investigators dichotomized their datasets right from the start of the analysis [10, 11, 20, 21]. We can show that this potentially biologically and clinically important phenomenon would also have been missed in our study had we used binary cut offs for analyses (Table 1).

Recent preclinical studies suggest that improved chemotherapy response in cancers with high intratumoural stroma content may be related to both, the structural components and protein products of the intratumoural stroma [23]. The presence of intratumoural stroma has been shown to modify the three-dimensional structure and composition of the tumour [26], which may result in an increase in interstitial fluid pressure, collapse of the microvasculature and reduction in tumour perfusion and chemotherapy drug delivery [24]. Furthermore, it has been suggested that tumour stroma produced proteins involved in vessel maturation and integrity such as VEGF, TFF-β, Ang1/2 and matrix metalloproteinases might result in the formation of a structurally and functionally abnormal tumour vasculature with areas of shunting and blind loops [27]. Hypoperfused tumour areas may a) not be reachable by chemotherapeutic drugs and b) may become hypoxic. Hypoxia has been suggested as one possible mechanism of cancer cell resistance to cytotoxic chemotherapy [28]. In addition, cancer associated fibroblasts have been shown to secrete fibronectin, connective tissue growth factor, hyaluron, matrix metalloproteinase and syndecan 1. These proteins have been suggested to induce cancer cell chemoresistance by reducing cell sensitivity to apoptosis and upregulating the expression of the multidrug resistance protein ABCB1 [23]. Contrastingly, however, tumour cells grown in cell culture in direct contact with each other (simulating the absence of stroma) have been shown to be more resistant to alkylating and platinum agents than the same cells after disaggregation [29, 30].

Based on our findings, we can currently only speculate that a fine balance between stroma abundance and tumour cell abundance might be necessary for chemotherapy to be most effective. Thus, patients may derive little or no benefit from chemotherapy if their cancers have a very high proportion of stroma (PoTlow) or very high proportion of tumour cells (PoThigh).

The ability of PoT to predict local tumour response (e.g. primary tumour regression) may be clinically useful for the pre-treatment assessment of borderline resectable cancers. However, we were unable to directly assess whether the response of the primary tumour was related to clinical downstaging of the tumour as detailed clinical and radiological staging data at the time of diagnosis were not collected in the OE02 trial [2]. Given the complexity and abundance of interactions between the intratumoural stroma and tumour cells in the presence of chemotherapy [23], further characterization of the tumour stroma components and their interaction with tumour cells is required to fully understand the biological mechanisms underpinning the findings of this study. Such studies may not only reveal basic principles of tumour cell and stroma interaction but may also identify new therapeutic targets in patients with both oesophageal and other epithelial cancers.

Limitations of our study include that we were only able to retrieve pre-treatment biopsy material from a subset of OE02 trial patients which was also related to the fact that at the time of the OE02 trial 129 (16%) patients were diagnosed by cytology only, or material was no longer present in the archive. Not all patients had matched resection specimens available, due to either the patient not undergoing surgery or material could not be retrieved. However, no difference was seen between the clinical and survival data of the patients who did and did not have biopsies available for analysis confirming that the investigated subgroup of patients was representative of the whole trial cohort. The amount of material available for assessment varied between different patients, a factor we compensated for by measuring biopsy pieces individually and adjusting the results by the size of the measurement area.

We are unable to directly validate the results from this study in a second independent phase 3 randomized trial cohort, as the OE02 trial changed clinical practice and there is no other trial in this patient population with a surgery alone control arm allowing to distinguish prognostic and predictive value of the biomarker.

In conclusion, this exploratory study is the first to demonstrate that proportion of tumour measurement in the diagnostic biopsies of patients with oesophageal cancer may be a clinically useful biomarker to stratify patients for treatment. Future studies into the tumour stroma content should use continuous measurement scales to allow non-linear associations to be detected. Further research is needed to refine the prediction model by detailed quantitative morphological and molecular characterization of the intratumoural stroma including its components to better understand the underlying biological processes and ultimately improve patient treatment stratification.

## MATERIALS AND METHODS

### Ethics statement

This study has been conducted in accordance with the ethical standards and according to the Declaration of Helsinki and according to national and international guidelines and was approved by the South East Research Ethics Committee, London, UK REC reference: 07/H1102/111.

### Patients

Eight hundred and two patients with microscopically or cytologically confirmed, previously untreated, resectable cancer of the oesophagus were included in the UK Medical Research Council (MRC) OE02 trial. Patients were randomized to treatment by surgery alone (S patients) or neoadjuvant combination chemotherapy consisting of two cycles 5-fluorouracil and cisplatin followed by surgery (CS patients) [2].

The Haematoxylin/Eosin (H&E) slide and/or blocks of the formalin fixed paraffin embedded diagnostic biopsies were retrospectively collected from 366 patients. Only tissue samples with a minimum of 0.04 mm^2^ tumour containing area were included in order to allow fitting of a minimum of 400 PoT measurement points. Samples with collections of malignant cells without intervening stroma, or no invasive malignancy were excluded. Material from 281 patients (140 CS patients and 141 S patients) fulfilled the inclusion criteria for the current study. Material retrieval and selection are shown in Figure 1.

### Clinicopathological data

Clinical data including age at randomization, cancer location (upper, middle or lower third of oesophagus), degree of dysphagia and patient performance status were collected during the clinical trial [2]. H&E slides from the biopsy and resection specimens were centrally reviewed at the time of slide and block collection to determine the histological tumour type, poorest grade of differentiation, tumour regression grading according to Mandard *et al*, [31] depth of invasion and lymph node status according to the Union for International Cancer Control TNM classification 6^th^ ed [32]. Histopathological data that were not assessable on review of the slide, such as tumour size, tumour location or number of lymph nodes were extracted from the original pathology report.

### Morphometric analysis of the biopsy and calculation of the proportion of tumour

The tumour and intratumoural stroma content was quantified using point counting with random systematic tissue sampling, a technique well-established for morphometric object quantification [33–36]. The original H&E stained diagnostic biopsy slides were used whenever possible. If the original slide was not available, 4 μm thick sections were cut from the biopsy paraffin block and stained with H&E using a standard protocol. For 35 patients, tumour cells were extremely difficult to identify on the H&E section and an additional slide was subjected to immunohistochemical staining with an epithelial cell marker (pan-cytokeratin, clone#:AE1/AE3 (Dako Cytomation), 1:100) using a routine protocol. Cytokeratin stained slides were quantified using the same protocol as for H&E stained slides.

All slides were scanned at 40x magnification using an Aperio XT scanner (Aperio Technologies, Vista, CA, USA) and examined using digital slide viewer software (ImageScopev11.1.2.752, Aperio Technologies, Vista, CA, USA). Regions with invasive tumour were identified and outlined using a tablet and pen tool (Cintiq 21UX LCD tablet, DTZ-2100D, Wacom). The software calculated automatically the size of the individually outlined region of interest. Areas consisting of necrosis, normal tissue or dysplasia were excluded. For the 837 regions identified, a random systematic grid of 600 measurement points ±5% tolerance was generated within the outline using virtual graticule software (RandomSpot, University of Leeds, Leeds, UK), [36]. Only one region was too small to fit 600 points and was therefore fitted with 400 measurement points. Each measurement point was manually reviewed and the tissue category underlying the measurement point was classified as tumour (T), intratumoural stroma (St: stroma, vessel, inflammation, or muscle) or non-informative (necrosis, extracellular mucin, normal tissue, keratin or non-classifiable due to artefacts). The method of outlining, superimposed grid and point counting is illustrated in Supplementary Figure S3.

Proportion of tumour cells (PoT) per case was calculated as the mean PoT of all biopsy pieces. To account for different sizes of the tissue regions, the PoT value per patient was normalized by the size of the measurement region so that the PoT per region was established as: ((number of points classified as T/number of points classified as either T or St)X(area of biopsy piece/total area of biopsy tissue per patient))x100.

Interobserver variation of the morphometric measurement was investigated by doublescoring a randomly selected sample of 10% of tissue pieces by a second independent histopathologist. Interobserver variation was assessed using Cohen's Kappa co-efficient (κ). The limits of agreement between observers were calculated using Bland-Altman plots. [25]

### Statistical methods

To assess whether the patient subset included in the study is representative of the whole OE02 trial population, patient characteristics (gender, site of tumour, histology, age, resection status (whether a resection was macro/microscopically complete) and tumour size) were compared between patients with and without PoT data.

The primary outcome measure for assessing the prognostic and predictive value of PoT was overall survival (time from randomization until death or last follow-up). Disease free survival was not used as an endpoint due to the absence of accurate data on oncological completeness of patient resections and uncertainty regarding the assessment methods used to diagnose disease recurrence. Data are presented graphically using Kaplan-Meier plots, and comparisons were made using log-rank tests and hazard ratios. Treatment interaction was assessed by calculating the heterogeneity of treatment effect across groups using a partial likelihood ratio test. Cox regression models included treatment, PoT, and interaction terms.

Tumour regression of the primary tumour in the resection specimen after chemotherapy was graded according to Mandard *et al*. [31] and compared with the pre-treatment biopsy PoT.

Categorical data were summarized using counts and percentages and continuous data using medians, ranges and inter-quartile ranges. Comparisons were made using chi-square, Kruskal-Wallis, Wilcoxon test or correlation coefficients as appropriate. As the analyses were hypothesis generating, no adjustments were made to account for multiple testing. Analyses were conducted using Stata Statistical Software: Release 12, College Station, TX: StataCorp LP or SPSS v.19, IBM Company, New York, New York State, USA.

To assess the prognostic and predictive value of the pre-treatment biopsy PoT, the data were analyzed using (1) PoT as a continuous variable, (2) a previously reported 50% cut off [20, 21] and (3) exploratory analyses to identify the optimal cut off by grouping patients by PoT values into three equally sized groups (PoT cut offs for three equal sized groups ≤49.15, 49.15 to 63.03 and ≥63.03). This initial analysis using tertiles suggested a greater benefit from chemotherapy in the middle tertile and no clear benefit to those in the upper and lower tertiles. To refine the cut off values, the data and relationship with survival were further explored by grouping patients into five equally sized groups (PoT cut offs for five equally sized groups: ≤41.86%, 41.86 to 51.39, 51.39 to 59.73, 59.73 to 69.38 and >69.38). Kaplan-Meier plots per treatment arm for the five equally sized groups are shown in Supplementary Figure S4. After visual assessment of the quintile Kaplan-Meier plots and with the aim to find a cut off that can relatively easily be assessed in future studies, patients were grouped as PoTlow (< 40%, *N=*46), PoTmedium (≥40% and ≤70%, *N=*183) and PoThigh (>70%, *N=*52).

The change in treatment effect across the full range of PoT values was further investigated using multivariable fractional polynomials, shown in Supplementary Figure 5.

The relationship between PoT and overall survival was examined within and between treatment arms.

A multivariate analysis including known prognostic factors such as depth of invasion and lymph node status as well as PoT class was found to be unfeasible as the PoT values were generated from the diagnostic (pre-treatment) biopsies and detailed pre-treatment staging data were not collected in this trial [2]. Using the pathological stage data after surgery was not an option as in patients treated with neoadjuvant chemotherapy, the stage could have been changed related to the chemotherapy.

## SUPPLEMENTARY MATERIALS FIGURES AND TABLES



## References

[1] Globocan (2008). Estimated cancer Incidence, Mortality, Prevalence and Disability-Adjusted Life Years (DALYs) Worldwide in 2008.

[2] Medical Research Council Oesophageal Cancer Working Party (2002). Surgical resection with or without preoperative chemotherapy in oesophageal cancer: a randomised controlled trial. Lancet.

[3] Van Heijl M, van Lanschot JJ, Koppert LB, van Berge Henegouwen MI, Muller K, Steyerberg EW, van Dekken H, Wijnhoven BP, Tilanus HW, Richel DJ, Busch OR, Bartelsman JF, Koning CC (2008). Neoadjuvant chemoradiation followed by surgery versus surgery alone for patients with adenocarcinoma or squamous cell carcinoma of the esophagus (CROSS). BMC Surg.

[4] Cunningham D, Allum WH, Stenning SP, Thompson JN, Van de Velde CJ, Nicolson M, Scarffe JH, Lofts FJ, Falk SJ, Iveson TJ, Smith DB, Langley RE, Verma M (2006). Perioperative chemotherapy versus surgery alone for resectable gastroesophageal cancer. N Engl J Med.

[5] Van Hagen P, Hulshof MC, van Lanschot JJ, Steyerberg EW, van Berge Henegouwen MI, Wijnhoven BP, Richel DJ, Nieuwenhuijzen GA, Hospers GA, Bonenkamp JJ, Cuesta MA, Blaisse RJ, Busch OR (2012). Preoperative chemoradiotherapy for esophageal or junctional cancer. N Engl J Med.

[6] Shapiro J, van Lanschot JJB, Hulshof MCCM, van Hagen P, van Berge Henegouwen MI, Wijnhoven BPL, van Laarhoven HWM, Nieuwenhuijzen GAP, Hospers GAP, Bonenkamp JJ, Cuesta MA, Blaisse RJB, Busch ORC (2012). Neoadjuvant chemoradiotherapy plus surgery versus surgery alone for oesophageal or junctional cancer (CROSS): long-term results of a randomised controlled trial. The Lancet Oncology.

[7] Dittrick GW, Weber JM, Shridhar R, Hoffe S, Melis M, Almhanna K, Barthel J, McLoughlin J, Karl RC, Meredith KL (2012). Pathologic nonresponders after neoadjuvant chemoradiation for esophageal cancer demonstrate no survival benefit compared with patients treated with primary esophagectomy. Annals of surgical oncology.

[8] Hanahan D, Coussens LM (2012). Accessories to the crime: functions of cells recruited to the tumor microenvironment. Cancer Cell.

[9] Yanagisawa N, Li R, Rowley D, Liu H, Kadmon D, Miles BJ, Wheeler TM, Ayala GE (2008). Stromogenic prostatic carcinoma pattern (carcinomas with reactive stromal grade 3) in needle biopsies predicts biochemical recurrence-free survival in patients after radical prostatectomy. Hum Pathol.

[10] De Kruijf EM, Van Nes JGH, van de Velde CJH, Putter H, Smit VTHBM, Liefers GJ, Kuppen PJK, Tollenaar RaEM, Mesker WE (2011). Tumor-stroma ratio in the primary tumor is a prognostic factor in early breast cancer patients, especially in triple-negative carcinoma patients. Breast Cancer Res Treat.

[11] Mesker WE, Junggeburt JMC, Szuhai K, de Heer P, Morreau H, Tanke HJ, Tollenaar RaEM (2007). The carcinoma-stromal ratio of colon carcinoma is an independent factor for survival compared to lymph node status and tumor stage. Cell Oncol.

[12] Nakajima I (1991). Immunohistochemical study of the extracellular matrix in non-small cell lung cancer: relation to lymph node metastasis and prognosis. Hokkaido Igaku Zasshi.

[13] Maeshima AM, Niki T, Maeshima A, Yamada T, Kondo H, Matsuno Y (2002). Modified scar grade: a prognostic indicator in small peripheral lung adenocarcinoma. Cancer.

[14] Schauer IG, Sood AK, Mok S (2011). Cancer-associated fibroblasts and their putative role in potentiating the initiation and development of epithelial ovarian cancer. Neoplasia.

[15] Athavale R, Thomakos N, Godfrey K, Kew F, Cross P, de Barros Lopes A, Hatem MH, Naik R The effect of epithelial and stromal tumor components on FIGO stages III and IV ovarian carcinosarcomas treated with primary surgery and chemotherapy. Int J Gynecol Cancer.

[16] Neesse A, Michl P, Frese KK, Feig C, Cook N, Jacobetz Ma, Lolkema MP, Buchholz M, Olive KP, Gress TM, Tuveson Da (2011). Stromal biology and therapy in pancreatic cancer. Gut.

[17] Ayala G, Tuxhorn JA, Wheeler TM, Frolov A, Scardino PT, Ohori M, Wheeler M, Spitler J, Rowley DR (2003). Reactive stroma as a predictor of biochemical-free recurrence in prostate cancer. Clin Cancer Res.

[18] Breuninger H, Schaumburg-Lever G, Holzschuh J, Horny HP (1997). Desmoplastic squamous cell carcinoma of skin and vermilion surface: a highly malignant subtype of skin cancer. Cancer.

[19] Otsuki S, Inokuchi M, Enjoji M, Ishikawa T, Takagi Y, Kato K, Yamada H, Kojima K, Sugihara K (2011). Vimentin expression is associated with decreased survival in gastric cancer. Oncol Rep.

[20] Courrech Staal EFW, Wouters MWJM, van Sandick JW, Takkenberg MM, Smit VTHBM, Junggeburt JMC, Spitzer-Naaykens JMJ, Karsten T, Hartgrink HH, Mesker WE, Tollenaar RaEM (2010). The stromal part of adenocarcinomas of the oesophagus: does it conceal targets for therapy?. Eur J Cancer.

[21] Courrech Staal EFW, Smit VTHBM, van Velthuysen M-LF, Spitzer-Naaykens JMJ, Wouters MWJM, Mesker WE, Tollenaar RaEM, van Sandick JW (2011). Reproducibility and validation of tumour stroma ratio scoring on oesophageal adenocarcinoma biopsies. Eur J Cancer.

[22] Wang K, Ma W, Wang J, Yu L, Zhang X, Wang Z, Tan B, Wang N, Bai B, Yang S, Liu H, Zhu S, Cheng Y (2012). Tumor-stroma ratio is an independent predictor for survival in esophageal squamous cell carcinoma. J Thorac Oncol.

[23] Hale MD, Hayden JD, Grabsch HI (2013). Tumour-microenvironment interactions: role of tumour stroma and proteins produced by cancer-associated fibroblasts in chemotherapy response. Cell Oncol (Dordr).

[24] Provenzano Pp Fau, Cuevas C, Cuevas C Fau, Chang AE, Chang Ae Fau, Goel VK, Goel Vk Fau, Von Hoff DD, Von Hoff Dd Fau, Hingorani SR, Hingorani SR (2012). Enzymatic targeting of the stroma ablates physical barriers to treatment of pancreatic ductal adenocarcinoma. Cancer Cell.

[25] Bland JM, Altman DG (1986). Statistical methods for assessing agreement between two methods of clinical measurement. Lancet.

[26] Tredan O, Galmarini CM, Patel K, Tannock IF (2007). Drug resistance and the solid tumor microenvironment. J Natl Cancer Inst.

[27] Goel S, Duda DG, Xu L, Munn LL, Boucher Y, Fukumura D, Jain RK (2011). Normalization of the Vasculature for Treatment of Cancer and Other Diseases. Physiological Reviews.

[28] Teicher BA (1996). A systems approach to cancer therapy. Cancer and Metastasis Reviews.

[29] Kerbel RS, St Croix B, Florenes VA, Rak J (1996). Induction and reversal of cell adhesion-dependent multicellular drug resistance in solid breast tumors. Human cell.

[30] Teicher BA, Herman TS, Holden SA, Wang YY, Pfeffer MR, Crawford JW, Frei E (1990). Tumor resistance to alkylating agents conferred by mechanisms operative only in vivo. Science (New York, NY).

[31] Mandard AM, Dalibard F, Mandard JC, Marnay J, Henry-Amar M, Petiot JF, Roussel A, Jacob JH, Segol P, Samama G (1994). Pathologic assessment of tumor regression after preoperative chemoradiotherapy of esophageal carcinoma. Clinicopathologic correlations. Cancer.

[32] Intenational Union Against C (2010). TNM: Classification of Malignant Tumours.

[33] Treanor D, Dattani M, Quirke P, Grabsch H (2008). Systematic random sampling with virtual slides: a new software tool for tissue research. J Pathol.

[34] West NP, Dattani M, McShane P, Hutchins G, Grabsch J, Mueller W, Treanor D, Quirke P (2010). The proportion of tumour cells is an independent predictor for survival in colorectal cancer patients. Br J Cancer.

[35] Treanor D, Dattani M, Quirke P, Grabsch H (2008). Systematic Random Sampling with Virtual Slides: A New Software Tool For Tissue Research.

[36] Wright A, Grabsch H, Treanor D RandomSpot: a web-based tool for systematic random sampling of virtual slides. J Pathol Inform.

[37] Royston P, Sauerbrei W (2009). Two techniques for investigating interactions between treatment and continuous covariates in clinical trials. Stata Journal.

[38] Royston P, Sauerbrei W (2004). A new approach to modelling interactions between treatment and continuous covariates in clinical trials by using fractional polynomials. Statistics in Medicine.

