# Supplementary material for: Biopsy proportion of tumour predicts pathological tumour response and benefit from chemotherapy in resectable oesophageal carcinoma: results from the UK MRC OE02 trial
Source: Oncotarget. 2016 Oct 18;7(47):77565–75. doi: 10.18632/oncotarget.12723 (PMC5363605; doi:10.18632/oncotarget.12723)
Supplement: Supplementary file 1 [file oncotarget-07-77565-s001.pdf]

Biopsy proportion of tumour predicts pathological tumour response and benefit from chemotherapy in resectable oesophageal carcinoma: results from the UK MRC OE02 trial

SUPPLEMENTARY FIGURES AND TABLES

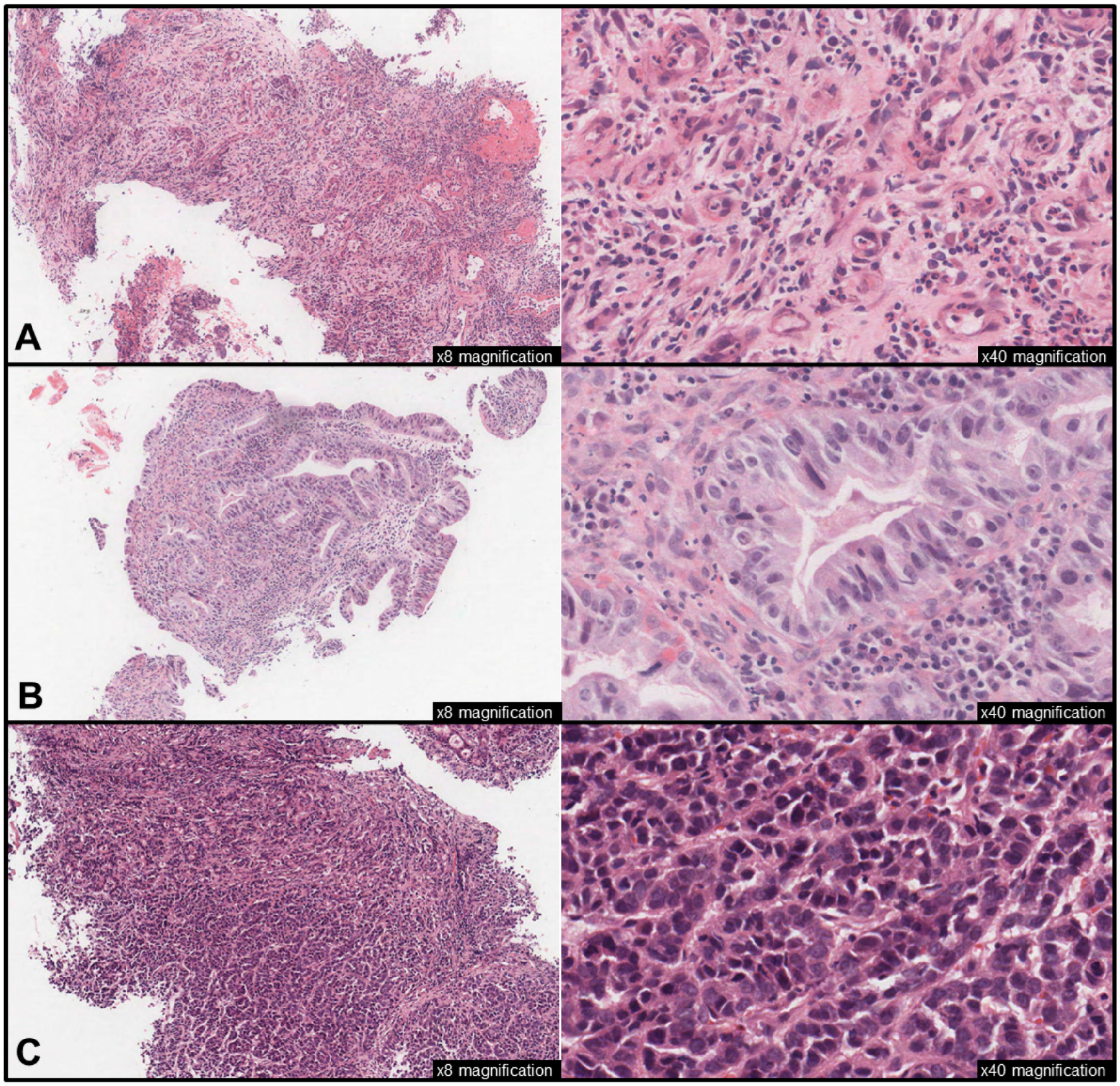

Supplementary Figure S1: Representative images of tumours classified as a proportion of tumour (PoT) of <40%, 40-70% and >70%. A. Adenocarcinoma classified as <40% tumour (exact PoT: 24.8%). **B.** Adenocarcinoma classified as 40%-70% tumour (exact PoT: 59.2%). **C.** Adenocarcinoma classified as >70% tumour (exact PoT: 89.6%)

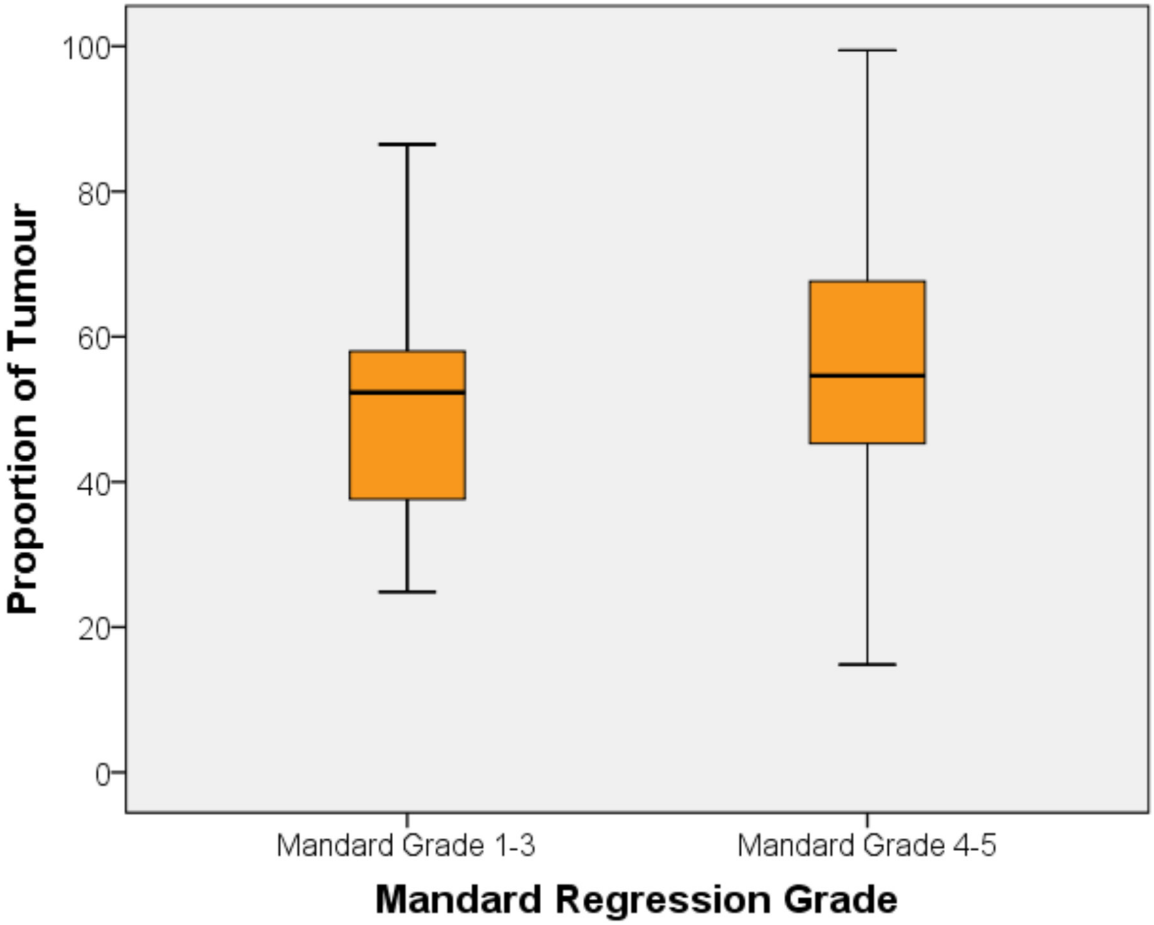

Supplementary Figure S2: Boxplot demonstrating the median, interquartile range and range of the proportion of tumour among patients with and without evidence of tumour regression (Mandard tumour regression grades 1-3 and 4-5, respectively).

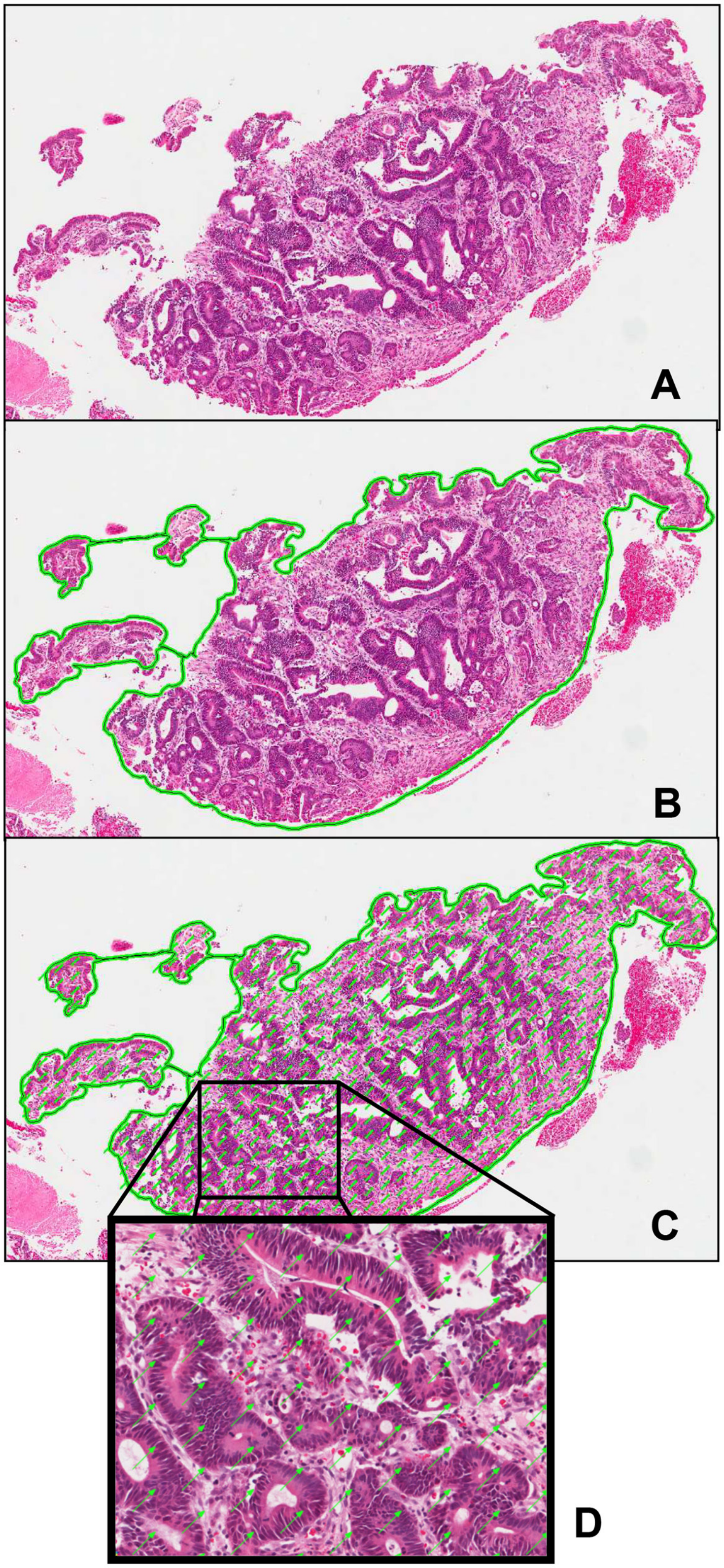

Supplementary Figure S3: Illustration of the point counting method. **A.** Selection of a biopsy piece with tumour. **B.** Outlining of the area of interest using a pen tool (green line). **C** and **D.** A grid with 600 equidistant measurement points generated using Randomspot software is superimposed onto the tissue section. The observer inspects all measurement points (tip of the arrow) manually and classifies the tissue type at this location.

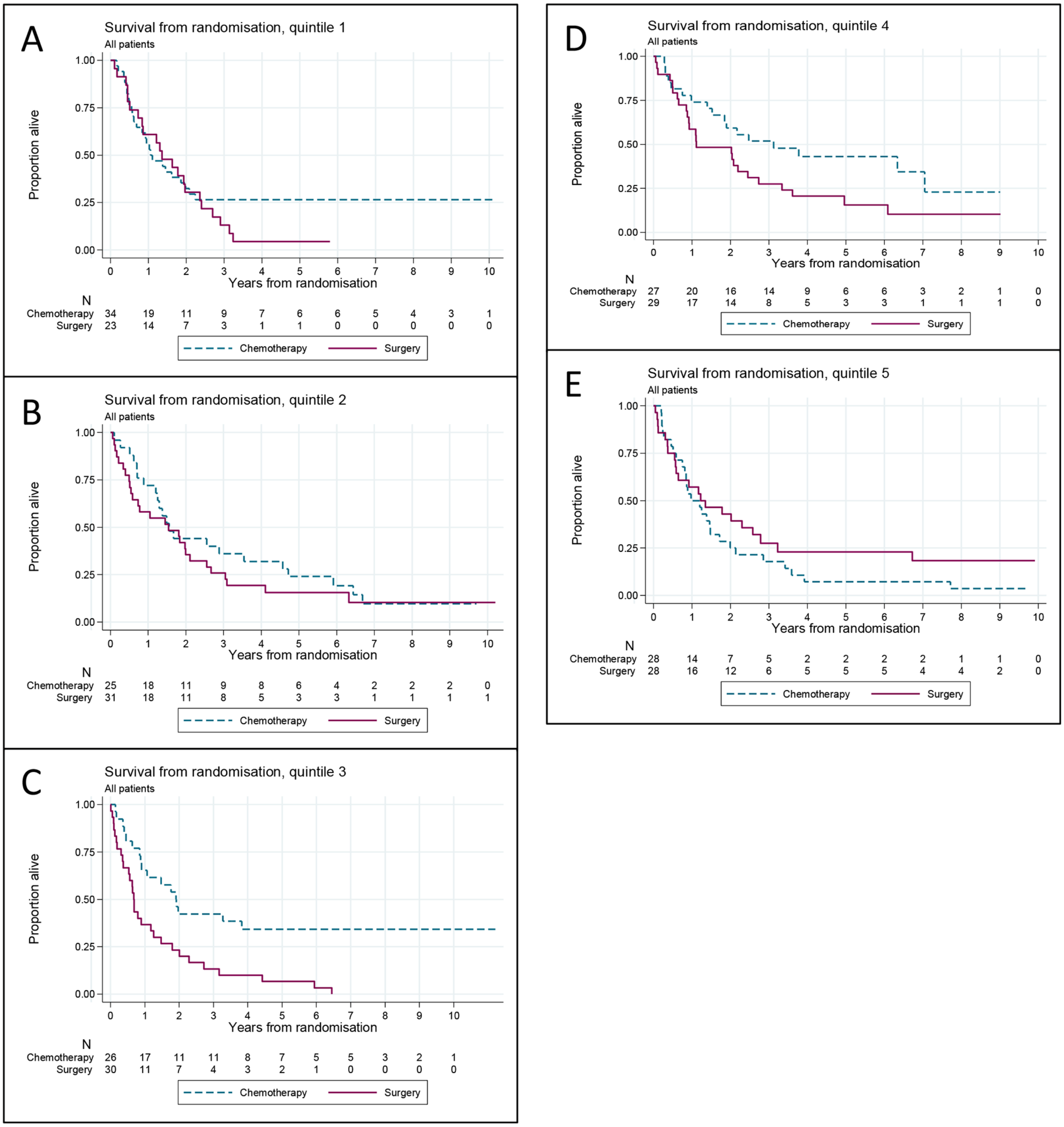

Supplementary Figure S4: Relationship between treatment arm and overall survival by proportion of tumour (study cohort divided into 5 equally sized groups (quintiles). A. biopsy proportion of tumour ≤41.86%: no significant difference in overall survival between treatment arms, HR (95% confidence interval): 1.31 (0.74, 2.33), p=0.3602. **B.** biopsy proportion of tumour between 41.86% and 51.39%: no significant difference in overall survival between treatment arms, HR (95% confidence interval): 1.29 (0.73, 2.27), p=0.3803. **C.** biopsy proportion of tumour between 51.39% and 59.73%: patients treated with neoadjuvant combination therapy followed by surgery survive significantly longer than those treated by surgery alone, HR (95% confidence interval): 2.64 (1.44, 4.84), p=0.0011. **D.** biopsy proportion of tumour between 59.73% and 69.38%: patients treated with neoadjuvant combination therapy followed by surgery survive significantly longer than those treated by surgery alone, HR (95% confidence interval):1.87 (1.00, 3.49), p=0.0457. **E.** biopsy proportion of tumour >69.38%: no significant difference in overall survival between treatment arms, HR (95% confidence interval): 0.73 (0.41, 1.27), p=0.2573.

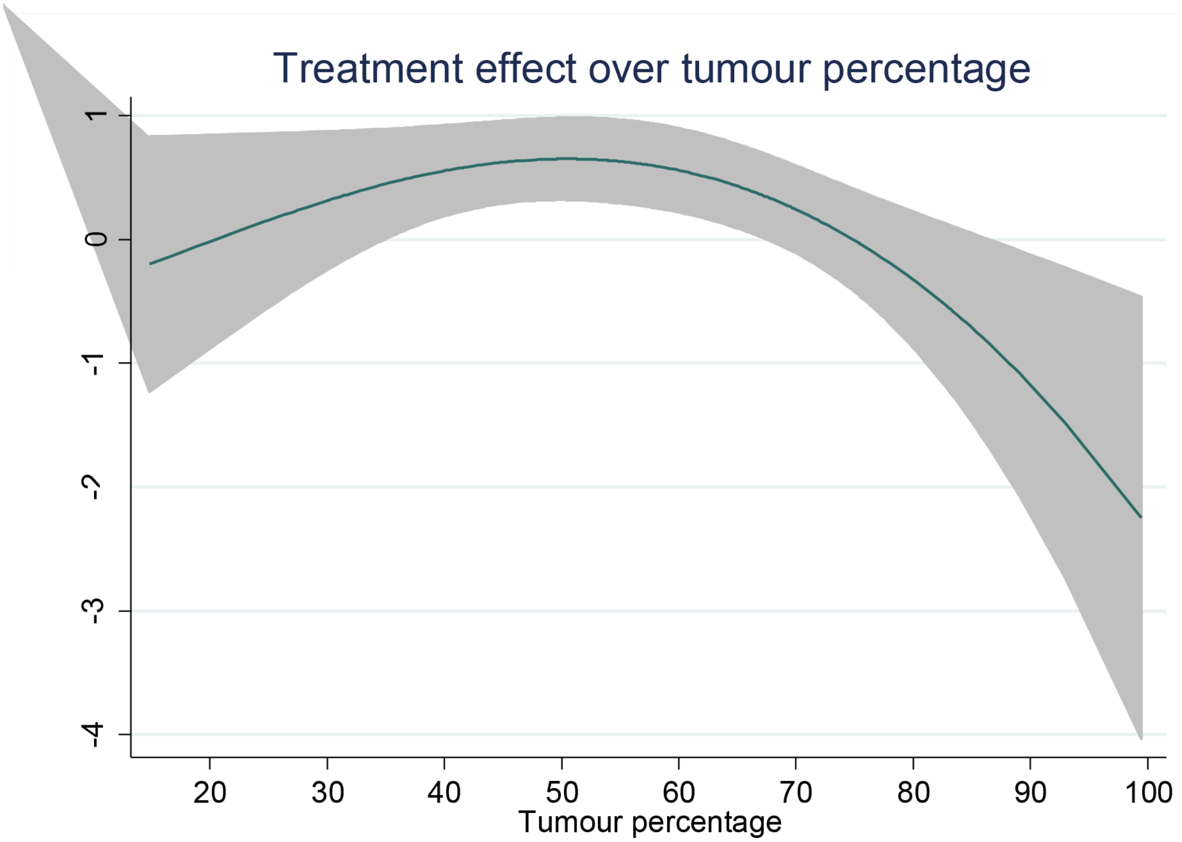

Supplementary Figure S5: The change in treatment effect according to the proportion of tumour. The central line represents the estimate of the log-hazard ratio, whilst the shaded area represents the 95% confidence interval around the estimate. The log-hazard value of 0 is equivalent to a hazard ratio of 1 (no difference between the treatment arms). Values above zero indicate a positive effect of chemotherapy on survival.

Supplementary Table S1: Comparison of baseline patient characteristics of the OE02 study cohort with the whole OE02 trial cohort

		Study cohort (*N*=281)	OE02 trial cohort (*N*=802)
Age (years)	Median	62	63
	IQR	54 – 69	55 – 68
	Range	30 – 83	30 – 84
		**n (%)**	**n (%)**
Sex	Male	213 (76)	603 (75)
	Female	68 (24)	199 (25)
Tumour site	Upper third	3 (1)	7 (1)
	Middle third	66 (23)	199 (25)
	Lower third	191 (68)	514 (64)
	Cardia	21 (7)	82 (10)
Histology type	Adenocarcinoma	195 (69)	533 (66)
	Squamous cell caricnoma	84 (30)	247 (31)
	Other	2 (1)	21 (3)
*Degree of dysphagia	0	41 (15)	108 (14)
	1	109 (41)	318 (42)
	2	80 (30)	234 (31)
	3	32 (12)	86 (11)
	4	3 (1)	8 (1)
	missing data	16	48
WHO performance status	0	188 (67)	532 (66)
	1	82 (29)	247 (31)
	2	10 (4)	21 (3)
	3	1 (0)	1 (<1)
	4	0	1 (<1)

Abbreviations: CS: patients treated with chemotherapy followed by surgery;IQR: interquartile range; S: patients treated with surgery alone; WHO: World Health Organisation.
* as described in the original trial publication: Medical Research Council Oesophageal Cancer Working Party. Surgical resection with or without preoperative chemotherapy in oesophageal cancer: a randomised controlled trial. *Lancet* 2002; **359**(9319): 1727-33.

Supplementary Table S2: Comparison of baseline patient characteristics of the OE02 study cohort between proportion of tumour groups

		Study cohort (*N*=281)	PoT <40% (*N*=46)	PoT 40-70% (*N*=183)	PoT >70% (*N*=52)
Age (years)	Median	62	62	62	64.5
	IQR	54 – 69	56 – 70	53 – 68	57 – 69
	Range	30 – 83	36 – 83	30 – 80	44 – 77
		**n (%)**	**n (%)**	**n (%)**	**n (%)**
Sex	Male	213 (76)	36 (78)	139 (76)	38 (73)
	Female	68 (24)	10 (22)	44 (24)	14 (27)
Tumour site	Upper third	3 (1)	1 (2)	2 (1)	0 (0)
	Middle third	66 (23)	8 (17)	42 (23)	16 (31)
	Lower third	191 (68)	36 (78)	122 (67)	33 (63)
	Cardia	21 (7)	1 (2)	17 (9)	3 (6)
Histology	Adeno	195 (69)	38 (83)	129 (70)	28 (54)
	Squamous	84 (30)	7 (15)	53 (29)	24 (46)
	Other	2 (1)	1 (2)	1 (1)	0 (0)
*Degree of dysphagia	0	41 (15)	6 (14)	30 (17)	5 (10)
	1	109 (41)	12 (28)	74 (43)	23 (48)
	2	80 (30)	15 (35)	49 (28)	16 (33)
	3	32 (12)	10 (23)	19 (11)	3 (6)
	4	3 (1)	0 (0)	2 (1)	1 (2)
	Data unavailable	16	3	9	4
WHO performance status	0	188 (67)	27 (59)	127 (69)	34 (65)
	1	82 (29)	15 (33)	52 (28)	15 (29)
	2	10 (4)	3 (7)	4 (2)	3 (6)
	3	1 (0)	1 (2)	0 (0)	0 (0)
	4	0	0 (0)	0 (0)	0 (0)

Abbreviations: PoT: Proportion of tumour; IQR: interquartile range; WHO: World Health Organisation. * The degree of dysphagia was classified as 0: Able to swallow all solids without difficulty, 1: Difficulty with swallowing some hard solids or particular foods, 2: Able to swallow a semi-solid or liquid diet only, 3: Able to swallow a liquid diet only and 4: Unable to swallow liquids or saliva

Supplementary Table 3: Relationship of proportion of tumour group and overall survival by treatment arm (patients with adenocarcinoma only)

	Pre-op chemotherapy followed by surgery			Surgery alone		
	PoT <40%	PoT 40% - 70%	PoT >70%	PoT <40%	PoT 40% - 70%	PoT >70%
Patients (*N*)	24	61	13	14	68	15
Events (*N*)	18	46	13	14	61	12
Median survival (years)	1·03	1·91	1·20	1·21	1·10	1·78
Log-rank *P*-value		0·0510			0·2134	
HR	REF	0·76	1·61	REF	0·75	0·49
HR *P*-value		0·323	0·193		0·345	0·083

Abbreviations: HR: hazard ratio; PoT: proportion of tumour
